# Assessing the direct occupational and public health impacts of solar radiation management with stratospheric aerosols

**DOI:** 10.1186/s12940-016-0089-0

**Published:** 2016-01-19

**Authors:** Utibe Effiong, Richard L. Neitzel

**Affiliations:** Department of Environmental Health Sciences, University of Michigan, 1415 Washington Heights, Ann Arbor, MI 48109 USA

**Keywords:** Climate change, Geoengineering, Solar radiation management, Aerosols, Exposure, Human health

## Abstract

Geoengineering is the deliberate large-scale manipulation of environmental processes that affects the Earth’s climate, in an attempt to counteract the effects of climate change. Injecting sulfate aerosol precursors and designed nanoparticles into the stratosphere to (i.e., solar radiation management [SRM]), has been suggested as one approach to geoengineering. Although much is being done to unravel the scientific and technical challenges around geoengineering, there have been few efforts to characterize the potential human health impacts of geoengineering, particularly with regards to SRM approaches involving stratospheric aerosols. This paper explores this information gap. Using available evidence, we describe the potential direct occupational and public health impacts of exposures to aerosols likely to be used for SRM, including environmental sulfates, black carbon, metallic aluminum, and aluminum oxide aerosols. We speculate on possible health impacts of exposure to one promising SRM material, barium titanate, using knowledge of similar nanomaterials. We also explore current regulatory efforts to minimize exposure to these toxicants. Our analysis suggests that adverse public health impacts may reasonably be expected from SRM via deployment of stratospheric aerosols. Little is known about the toxicity of some likely candidate aerosols, and there is no consensus regarding acceptable levels for public exposure to these materials. There is also little infrastructure in place to evaluate potential public health impacts in the event that stratospheric aerosols are deployed for solar radiation management. We offer several recommendations intended to help characterize the potential occupation and public health impacts of SRM, and suggest that a comprehensive risk assessment effort is needed before this approach to geoengineering receives further consideration.

## Background

Warming of the climate system is unequivocal, and since the 1950s, human influence on the climate system has become clear [1, 2]. Because human activities have become significant geological forces, the term “anthropocene” has been applied to the current geological epoch, which began in the eighteenth century [3]. The United Nation’s Intergovernmental Panel on Climate Change (IPCC) has forecast that if human activity and world development continue unimpeded, average surface temperatures could rise as much as 4.8 °C by 2100 [1, 2, 4]. The lack of success to date in efforts to reduce greenhouse gas emissions sufficiently has prompted attention to the possibility of counteracting the effects of emissions through the intentional manipulation of global-scale Earth system processes – a process referred to as “geoengineering” [5]

**Fig. 1:**
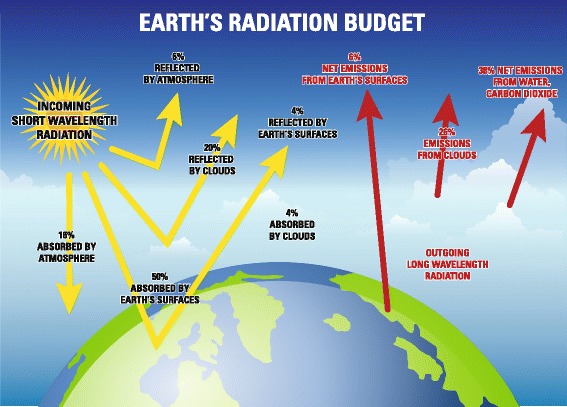
Components of the earth’s radiation budget (adapted from NASA. http://science-edu.larc.nasa.gov/EDDOCS/whatis.html)

**Fig. 2:**
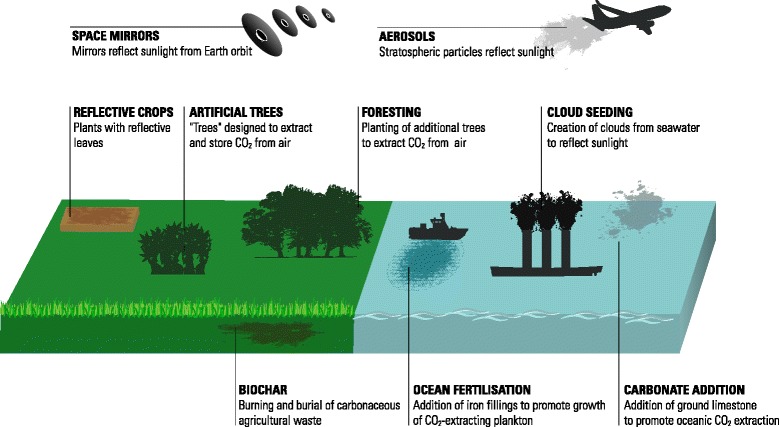
Potential methods for solar radiation management and carbon dioxide removal (adapted from http://r3zn8d.files.wordpress.com/2013/04/geoengineering.jpg)

The concept of geoengineering is not new, and dates back to at least 1965 [6]. However, the term geoengineering as applied in its current context was introduced in 1977 [7]. Geoengineering approaches include solar radiation management, or SRM, and carbon dioxide removal (CDR) [5]. SRM techniques attempt to offset effects of increased greenhouse gas concentrations by reducing the proportion of incoming short wavelength solar radiation that is absorbed or reflected by the earth’s atmosphere (Fig. 1) [8]. Proposed SRM techniques include stratospheric aerosols, reflective satellites, whitening of the clouds, whitening of built structures and increasing plant reflectivity (Fig. 2) [5]. All SRM deployment techniques require a global approach since localized deployment will not produce sufficient effects. Importantly, SRM approaches to managing climate change require initial and ongoing addition of aerosols to the atmosphere, with increasingly greater additions as emissions of GHGs rise, given the risk of sudden and potentially catastrophic warming if aerosol levels are not maintained. Proposed CDR approaches include afforestation/reforestation, direct air carbon dioxide (CO_2_) capture/storage, manufacturing carbonate minerals using silicate rocks and CO_2_ from the air, accelerated weathering of rocks, ocean alkalinity addition and ocean fertilization (Fig. 2) [5].

This paper will focus on SRM via stratospheric aerosol injection, and will describe potential direct human health impacts. We explore three knowledge gaps: 1) human exposures, 2) human health impacts, and 3) exposure limits. SRM may be expected to result in ecosystem damage and resulting human health effects through indirect mechanisms such as damage to, or contamination of, agricultural products and wildlife. While these effects are important, they are beyond the scope of our paper.

### Stratospheric aerosols for use in SRM

The stratosphere is the second major layer of Earth’s atmosphere, lying immediately above the lowest layer (the troposphere) at an altitude of 10–50 km [9]. Within the stratosphere temperatures increase with increasing elevation. The potential for SRM from stratospheric injection of aerosols has been demonstrated by global cooling following large volcanic eruptions [10].

A wide range of particles could be released into the stratosphere to achieve the SRM objective of scattering sunlight back to space. Sulfates and nanoparticles currently favored for SRM include sulfur dioxide, hydrogen sulfide, carbonyl sulfide, black carbon, and specially engineered discs composed of metallic aluminum, aluminum oxide and barium titanate [11]. In particular, engineered nanoparticles are considered very promising. The particles would utilize photophoretic and electromagnetic forces to self-levitate above the stratosphere [11]. These nanoparticles would remain suspended longer than sulfate particles, would not interfere with stratospheric chemistry, and would not produce acid rain [12]. However, while promising, the self-levitating nanodisc has not been tested to verify efficacy, may increase ocean acidification due to atmospheric CO_2_ entrapment, has uncharacterized human health and environmental impacts, and may be prohibitively expensive [12].

### Knowledge gap 1: human exposures

Human exposures to materials used for SRM could occur during the manufacture, transportation, deployment and post-deployment of these materials [13]. In this paper, unless otherwise stated, inhalation is the primary route of exposure considered.

#### Occupational exposures

Airborne sulfate exposures have been shown to range up to 23 mg/m^3^ in sulfuric acid plants [14]. Additionally, high exposures to sulfuric acid fumes have also been noted in the petrochemical industry, and high exposures to hydrogen sulfide and carbonyl sulfide have also been noted in natural gas extraction operations [15, 16]. Exposures to black carbon during its manufacture can be quite high [17]. Elevated airborne exposures to aluminum and its oxide have been shown to occur during aluminum refining, smelting and at aluminum powder plants [18]. There appears to be no available documentation of occupational exposure to barium titanate. In addition to manufacturing settings, exposures to SRM materials could occur during deployment, e.g., during cloud seeding operations, as well as from accidents during transportation [19, 20].

Occupational exposures to SRM materials are likely to occur over brief periods (e.g., days to weeks), with the potential for repeated or cyclic exposures. The health effects of such exposures will therefore likely be acute in nature, though repeated exposures create an opportunity for chronic health effects. Occupational exposures may be attenuated through the use of engineering controls such as ventilation, as well as the use of personal protective equipment (PPE) such as respirators and protective suits.

#### Population exposures

Due to atmospheric circulation and gravitational deposition, large-scale population exposures to atmospherically-injected SRM materials will almost certainly occur after their deployment. Population exposures could also occur through ingestion of food and water contaminated with deposited particles, as well as transdermally [11, 21]. Unlike occupational exposures, there has been virtually no research done to estimate ground-level personal exposures to SRM materials, though the US Environmental Protection Agency (EPA) does provide guidance on methods for evaluating environmental exposures to several possible SRM materials [22].

Stratospheric injection of sulfur dioxide and black carbon has already been modeled to analyze potential deposition of sulfate and soot [21, 23]. One model estimated that with 1 Tg of black carbon infused into the stratosphere annually, after ten years of geoengineering, the globally averaged mass burden would be approximately 8 × 10^−6^ kg m^−2^ [23]. The intentional addition of black carbon to the atmosphere will exacerbate adverse health effects already resulting from unintentional release at ground level [24]. In the year 2000, the global emission of black carbon was estimated at 7.6 Tg, and the globally averaged mass burden of black carbon was roughly 1.5 × 10^−5^ kg m^−2^ [25]. No models appear to have estimated the potential global burden of environmental aluminum, alumina or barium titanate that might result from SRM.

In contrast to occupational exposures, population exposures to SRM materials will be continuous and prolonged over months to years, but will likely be orders of magnitude lower than those experienced occupationally. Thus the health effects will be primarily chronic in nature. The use of PPE to reduce personal exposures to deposited SRM materials is not feasible on a population scale.

### Knowledge gap 2: potential human health impacts

Table 1 summarizes, by bodily system, the potential human health effects of the aerosols that may be used for SRM.

**Table 1:** Human health effects of the potential SRM aerosols

	Potential SRM aerosol						
Health effect/target system	Sulfuric acid	Sulfur dioxide	Hydrogen sulfide	Carbonyl sulfide	Black carbon	Aluminum compounds	Barium compounds
Respiratory	X	X	X	X	X	X	X
Cardiovascular	X	X	X	X	X	X	-
G.I	-	X	X	X	-	-	X
Hematologic	-	X	X	X	X	X	-
Musculosketal	-	-	X	X	-	X	X
Hepatic	-	-	-	-	X	-	-
Renal	-	-	-	X	-	-	X
Endocrine	-	-	-	-	-	X	-
Dermal	X	X	X	X	-	-	-
Ocular	X	X	X	X	X	-	-
Metabolic	X	-	X	X	-	-	X
Immunologic	-	X	-	-	-	X	-
Neurologic	X	X	X	X	X	X	X
Reproductive	-	X	X	-	-	-	-
Developmental	-	X	-	-	-	-	-
Genotoxic	-	X	-	-	-	-	-
Cancer	X	-	-	-	X	X	-
Death		X	X	X	-	X	X

*X* Data suggest health hazard possible, *-* insufficient data available

Inhalational studies with sulfuric acid aerosol suggest that it has a local irritant effect and no systemic effects [26]. Squamous cell metaplasia in the laryngeal epithelium has been observed in animal studies at exposures as low as 0.3 mg/m^3^, with more severe metaplasia following exposures of 1.38 mg/m^3^. Epidemiological studies suggest a relationship between exposure to mists containing sulfuric acid and an increased incidence of laryngeal cancer, and the International Agency for Research on Cancer has concluded that “occupational exposure to strong inorganic mists containing sulfuric acid is carcinogenic for humans” [27, 28].

In humans, and in particular asthmatics, increases in specific airway resistance or decreases in forced expiratory volume or forced expiratory flow are the primary response following acute exposure to sulfur dioxide [29]. Cough, irritation, increased salivation, and erythema of the trachea and main bronchi occurred following controlled exposures to ≤8 ppm for 20 min [30]. Exposures to higher levels (e.g., 40 ppm) can produce a burning sensation in the nose and throat, dyspnea, and severe airway obstruction that may only partially reverse over time [31]. Exposures to even higher levels (e.g., ≤100 ppm) can result in reactive airway dysfunction syndrome, which involves bronchial epithelial damage and increased sensitization and nonspecific hypersensitivity to other irritant stimuli [32, 33]. Deaths can occur following exposures >100 ppm [31].

Single exposures to hydrogen sulfide can cause health effects in many systems [34]. Hydrogen sulfide has an odor threshold of 0.01 mg/m^3^, and humans become insensitive to its odor at concentrations of ≥140 mg/m^3^ [35, 36]. Respiratory symptoms in asthmatic individuals appear at about 2.8 mg/m^3^, but respiratory distress does not seem to occur <560 mg/m^3^ [37]. Eye irritation can occur at 5–29 mg/m^3^, and metabolic abnormalities may occur at 7 mg/m^3^ [38]. Neurological symptoms such as fatigue, loss of appetite, headache, irritability, poor memory and dizziness may result following exposures >28 mg/m^3^ [39], with death occuring. > 700 mg/m^3^ [40].

Limited information is available on the pharmacokinetics of carbonyl sulfide, which likely metabolizes to carbon dioxide and hydrogen sulfide [41]. Acute exposures result in symptoms similar to those of hydrogen sulfide, but with less local irritation or olfactory warning [42]. Sublethal exposure can result in profuse salivation, headache, vertigo, amnesia, confusion, nausea, vomiting, diarrhea, cardiac arrhythmia, weakness, muscle cramps, and unconsciousness [43]. Concentrations >1000 ppm can cause sudden collapse, convulsions, and death from respiratory paralysis.

Respiratory effects in black carbon workers include cough, sputum production, bronchitis, pneumoconiosis, and decrements in lung function, as well as tiredness, chest pain, headache, and respiratory irritation [24, 44, 45]. Black carbon may cause discoloration of eyelids and conjunctivae [46], and is possibly carcinogenic to humans (Group 2B); there is inadequate evidence of carcinogenicity in humans, but sufficient evidence in experimental animals [24].

Aluminum is never found free in nature, and instead forms metal compounds, complexes, or chelates including aluminum oxide [47]. Aluminum and aluminum oxide do not appear to differ in toxicity [47]. Wheezing, dyspnea, and impaired lung function, as well as pulmonary fibrosis, have been noted in workers exposed to fine aluminum dust [48–50]. Dilation and hypertrophy of the right side of the heart have been seen in workers exposed to aluminum powder, as have decreased red blood cell hemoglobin and finger clubbing [50]. Helper T-lymphocyte alveolitis and blastic transformation of peripheral blood lymphocytes in the presence of soluble aluminum compounds in vitro were found in an individual exposed to aluminum dust [51]. There is limited evidence of carcinogenicity among workers; the few existing studies have been confounded by concurrent exposures to known carcinogens, (e.g., tobacco smoke or polycyclic aromatic hydrocarbons) [52].

Barium titanate is a complex salt containing two metals, which complicates modeling of its toxicological properties. In general, exposures to barium salts are associated with respiratory, cardiovascular, gastrointestinal, musculoskeletal, metabolic and neurologic effects [53]. Barium salts also have a local effect on skin surfaces and would not likely be absorbed systematically to any great extent, though this might not be true of barium salt nanoparticles [53, 54]. Barium titanate could also behave like a titanium salt in interactions with the human body, in which case the resulting health effects are essentially unknown. Only two titanium-containing compounds are indexed by the U.S Agency for Toxic Substances and Disease Registry (ATSDR) or covered by U.S exposure limits [55]. It is possible that barium titanate might act both as a salt of barium and titanium, or as neither; the toxicological properties of a nanoparticle are influenced by factors such as particle size, surface area, chemistry or reactivity, solubility, and shape [54].

### Knowledge gap 3: exposure standards and guidelines

Several US agencies and organizations have established occupational exposure limits (OELs) for sulfate, carbon, and some metallic substances. While OELs almost uniformly assume an 8-h daily exposure period, organizations use different assumptions and acceptable excess risk levels when establishing limits. As a result there are a range of OELs for potential SRM materials, which complicates the establishment of “safe” global levels. Additionally, some potential SRM compounds (for example, barium titanate) are currently unregulated and/or have no recognized occupational exposure assessment procedures. All of these issues apply equally to community exposure limits.

The American Conference of Governmental Industrial Hygienists (ACGIH) Threshold Limit Values (TLVs) for the potential SRM materials shown in Table 2 are consistently lower than those required by the U.S Occupational Safety and Health Administration (OSHA) or recommended by the U.S National Institute for Occupational Safety and Health (NIOSH) [56, 57] The TLVs and NIOSH Recommended Exposure Limits (RELs) are intended to protect the typical worker from any adverse health effects without consideration of economic or political feasibility, while the OSHA limits consider technical and economic feasibility and are subsequently less protective [56, 58].

**Table 2:** Occupational exposure standards for substances that may be utilized in solar radiation management (Unless otherwise specified, exposure limits are average levels over an 8-h workday)

Substance	U.S Occupational Safety and Health Administration (mg/m^3^)^a^	U.S National Institute for Occupational Safety and Health (mg/m^3^)^a^	American Conference of Governmental Industrial Hygienists (mg/m^3^)^a^
Sulfuric acid	1	1	0.2
Sulfur dioxide	13	5.2	0.7^b^
	--	13.1^b^	--
Hydrogen Sulfide	27.9^c^	13.9^c^	1.4
	60.7^d^	--	7.0^b^
Carbonyl Sulfide	-	-	12.3
Black carbon	3.5	3.5	3
Aluminum aerosol	15	10	1^e^
	5^e^	5^e^	--
Aluminum oxide	15	-	-
	5^e^	--	
Barium titanate	-	-	-

^a^. Computed from standards specified in parts per million

^b^. Short-term exposure limit (15 minutes)

^c^. Ceiling limit

^d^. 10-minute single period exposure limit

^e^. Respirable fraction

For public exposures – which would likely be widespread following SRM efforts – the EPA, European Environmental Agency (EEA), and World Health Organization specify regulatory standards for ambient air quality (Table 3) [57–59]. Importantly, Table 3 shows a very small sampling of air quality standards in use around the world that relate to potential SRM materials, of which the WHO standards may be considered most generalizable globally. Exposure limits differ substantially between these agencies, but, more importantly, there are currently no limits set by any of these agencies for most of the substances that may be used for SRM [60, 61].

**Table 3:** Ambient air quality standards for substances that may be utilized in solar radiation management

Substance	U.S Environmental Protection Agency		European Environmental Agency		World Health Organization	
	Limit (μg/m^3^)	Averaging period	Limit (μg/m^3^)	Averaging period	Limit (μg/m^3^)	Averaging period
Sulfuric acid	-	-	-	-	-	-
Sulfur dioxide	196.5	1 h	350	1 h	20	24 h
			125	24 h	500	10 min
Hydrogen sulfide	-	-	-	-	-	-
Carbonyl sulfide	-	-	-	-	-	-
Nanoparticles	-	-	-	-	-	-
PM_2.5_	12	1 year	25	1 year	10	1 year
	35	24 h	--	--	25	24 h

The inconsistencies in established exposure limits for both occupational and community settings, combined with the absence of any exposure limits for a number of potential SRM materials, highlight the issues involved in protecting workers and the public from unintended health consequences resulting from SRM deployment. Since employers have legal control over exposures to their workers, OELs can be met through implementation of engineering controls and use of PPE, whereas use of PPE is not feasible at a population level, and reductions in public exposures would have to rely on engineering controls (e.g., use of air cleaning devices) or administrative controls (e.g., behavior changes). The substantial potential exposures and subsequent health impacts associated with SRM efforts based on stratospheric aerosols must be considered further before any attempts are made at SRM .

### Recommendations

In order to be effective, SRM efforts involving stratospheric aerosols will require a global effort. Such an action would represent the first truly global and intentionally-produced human exposures, and because the benefits and potential consequences of this action would impact the entire population of the planet to some degree, we make the following initial recommendations:

i. Geoengineering cost-benefit analyses should consider health impacts of SRM.
  At present, most assessments of geoengineering are done within specific and well-defined frameworks of economics, risk, politics, and environmental ethics [62]. Literature on the potential human health impacts of SRM is scant, and such impacts have not been adequately factored into previous cost-benefit analyses [63]. We recommend that subsequent cost-benefit analyses for geoengineering explicitly consider health impacts of SRM [64]. Assessments should further compare the expected health benefits that may result from SRM efforts to potential adverse health outcomes, including (but not limited to) those described here.
ii. Further research is needed on methods of assessment of exposures to, and evaluation of toxicological properties of, potential SRM materials.
  We have noted gaps in current scientific knowledge related to occupational and community exposures that would result from SRM, as well to the toxicological properties of potential SRM materials. Additional laboratory- and field-based research is needed in these areas, particularly with regard to exposure characterization and the spatial and temporal movement of SRM materials from the stratosphere to ground level. While it is difficult to develop exposure and toxicological models which are representative of a decades- or centuries-long SRM deployment, these efforts are critical to ensure that reasonable, validated models of exposures and human health impacts are available prior to any SRM deployment.
iii. Strict and harmonized global occupational and community exposure limits are needed for SRM materials.
  Tables 2 and 3 illustrate the divergence and incompleteness of current occupational and community exposure limits regarding potential SRM materials. Since exposures will inherently be global in nature, exposure limits must be harmonized to ensure that individuals around the world are given equal protection from adverse health effects. Global harmonization of standards related to SRM represents an immense but necessary bureaucratic and scientific challenge, and an important step towards establishing a formal governance framework for geoengineering. A global discussion of standards harmonization relating to SRM may result in other tangible benefits to society, including the potential evolution of a common language and framework for risk assessment and a debate on the strengths and weaknesses of different approaches to risk management.
iv. Reversal mechanisms should be identified prior to any SRM deployment
  In the event that substantial health impacts are noted following deployment of stratospheric aerosol approaches to SRM, mechanisms for capturing the aerosols to halt further ground-level exposures through gravitational deposition will be needed. Therefore, if stratospheric aerosols are pursued as a viable SRM strategy, such mechanisms will need to be identified and evaluated prior to large-scale deployment.

## Conclusion

Although there is very little agreement in the scientific community on the approach to SRM-related technologies, SRM has been identified as a potentially technically feasible and possibly cost-effective method of geoengineering to reduce or reverse anthropogenically-driven climate change [1, 62]. But even as much is being done to unravel the scientific and technical challenges around geoengineering, and there is substantial evidence that a host of adverse human health effects will directly result from climate change, very little has been done to describe the potential human health impacts of this emerging disruptive technology. We have described the potential occupational and public health impacts of inadvertent exposure to potential SRM materials, and have also speculated on the possible health impacts of exposure to barium titanate using knowledge of similar nanomaterials.

Based on our analyses, we submit that the current knowledge gaps do not justify deployment of SRM in the short term. We therefore recommend further research, a more inclusive analysis of costs and benefits, as well as the globalization and harmonization of regulatory standards that will limit the negative human health impact of SRM. Only following a comprehensive risk assessment that addresses each of these issues can the potential benefits of this geoengineering approach be weighed against the potential public health burdens created by this technology.

## Abbreviations

ACGIH: American Conference of Governmental Industrial Hygienist
ATSDR: U.S Agency for Toxic Substances and Disease Registry
CDR: Carbon Dioxide Removal
EPA: U.S Environmental Protection Agency
IPCC: Intergovernmental Panel on Climate Change
NIOSH: National Institute for Occupational Safety and Health
OSHA: Occupational Safety and Health Administration
PPE: Personal Protective Equipment
REL: Recommended Exposure Limits
SRM: Solar Radiation Management
TLV: Threshold Limit Values

## References

[1] Stocker T, Qin D, Platner G (2013). Climate change 2013: the physical science basis. Intergovernmental panel on Climate Change, Working Group I. Contribution to the IPCC Fifth Assessment Report (AR5).

[2] Pachauri R, LA M. IPCC, 2014: Climate Change 2014: Synthesis Report. Contribution of Working Groups I, II and III to the Fifth Assessment Report of the Intergovernmental Panel on Climate Change. Geneva, Switzerland; 2015.

[3] Crutzen P, Ehlers E, Krafft T (2006). The “anthropocene.”. Earth system science in the anthropocene.

[4] Pachauri RK, Reisinger A (2008). Climate change 2007. Synthesis report. Contribution of working groups I, II and III to the fourth assessment report.

[5] Shepherd J (2009). Geoengineering the climate: science, governance and uncertainty.

[6] Revelle R, Broecker W, Craig H. Restoring the Quality of Our Environment: Report of the Environmental Pollution Panel. President's Science Advisory Committee, United States, US Government Printing Office: Washington, D.C. 1965.

[7] Marchetti C (1977). On geoengineering and the CO2 problem. Clim Change.

[8] Akbari H, Menon S, Rosenfeld A (2009). Global cooling: increasing world-wide urban albedos to offset CO2. Clim Change.

[9] Labitzke K, Van Loon H (1999). The stratosphere: phenomena, history, and relevance.

[10] Crutzen PJ (2006). Albedo enhancement by stratospheric sulfur injections: a contribution to resolve a policy dilemma?. Clim Change.

[11] Keith DW (2010). Photophoretic levitation of engineered aerosols for geoengineering. Proc Natl Acad Sci U S A.

[12] Resnik D, Vallero D. Geoengineering: An Idea Whose Time Has Come? J Earth Sci Clim Chang 2011(Suppl 1). PMC3596048 23502911

[13] Caldeira K, Bala G, Cao L (2013). The science of geoengineering. Annu Rev Earth Planet Sci.

[14] Wigley TML (2006). A combined mitigation/geoengineering approach to climate stabilization. Science.

[15] Soskolne C, Jhangri G (1992). Occupational exposure to sulfuric acid in southern Ontario, Canada, in association with laryngeal cancer. Scand J Work Environ Heal.

[16] Weiss C. Sour gas treatment process. US Patent 2012.

[17] Gardiner K, Calvert I (1996). Occupational exposure to carbon black in its manufacture: data from 1987 to 1992. Ann Occup Hyg.

[18] Letzel S, Lang C, Schaller K, Angerer J (2000). Longitudinal study of neurotoxicity with occupational exposure to aluminum dust. Neurology.

[19] Ng W, Koh D (2011). Occupational contact dermatitis in manual cloud seeding operations. Singapore Med J.

[20] Christiansen S, Urquhart S. Emergency Planning and Community Right to Know Act of (1986). Analysis and update. Brigham Young Univ J Public Law.

[21] Kravitz B, Robock A, Oman L. Sulfuric acid deposition from stratospheric geoengineering with sulfate aerosols. J Geophys Res Atmos 2009, 114.

[22] U.S Environmental Protection Agency. Air Science [http://www2.epa.gov/science-and-technology/air-science]. Accessed 2 Oct 2015.

[23] Kravitz B (2011). Stratospheric geoengineering with black carbon aerosols.

[24] Baan R, Straif K, Grosse Y (2006). Carcinogenicity of carbon black, titanium dioxide, and talc. Lancet Oncol.

[25] U.S Environmental Protection Agency. Report to Congress on Black Carbon. Report EPA-450/R-12-001. US Government Printing Office: Washington, DC; 2012.

[26] National Library of Medicine. Sulfuric Acid,CASRN. 7664-93-9 [http://toxnet.nlm.nih.gov/cgi-bin/sis/search/a?dbs+hsdb:@term+@DOCNO+1811]. Accessed 2 Oct 2015.

[27] Hernandez O, Heiskanen M (2000). SIDS initial assessment report for 11 Th SIAM.

[28] International agency for Research on Cancer (1992). Occupational exposures to mists and vapours from strong inorganic acids: and other industrial chemicals.

[29] Pohl H, Liccione J, Iannucci A (1998). Toxicological profile for sulfur dioxide.

[30] Sandström T, Stjernberg N (1989). Is the short term limit value for sulphur dioxide exposure safe? Effects of controlled chamber exposure investigated with bronchoalveolar lavage. Br J Ind Med.

[31] Rabinovitch S, Greyson N (1989). Clinical and laboratory features of acute sulfur dioxide inhalation poisoning: Two-year follow-up. Am Rev Respir Dis.

[32] Skalpe I (1964). Long-term effects of sulphur dioxide exposure in pulp mills. Br J Ind Med.

[33] Brooks S (2007). Occupational and environmental asthma. Environmental and occupational medicine. Fourth.

[34] Chou C (2003). Concise International Chemical Assessment Document 53: Hydrogen Sulfide: Human Health Aspects.

[35] Amoore J, Hautala E (1983). Odor as an ald to chemical safety: odor thresholds compared with threshold limit values and volatilities for 214 industrial chemicals in air and water dilution. J Appl Toxicol.

[36] Hirsch A, Zavala G (1999). Long-term effects on the olfactory system of exposure to hydrogen sulphide. Occup Environ Med.

[37] Jäppinen P, Kangas J, Silakoski L, Savolainen H (1993). Volatile metabolites in occupational exposure to organic sulfur compounds. Arch Toxicol.

[38] Lambert T, Goodwin V (2006). Hydrogen sulfide (H2S) and sour gas effects on the eye. A historical perspective. Sci Total Environ.

[39] Reiffenstein R (1992). Toxicology of hydrogen sulfide. Annu Rev Pharmacol Toxicol.

[40] Beauchamp R, Bus J (1984). A critical review of the literature on hydrogen sulfide toxicity. CRC Crit Rev Toxicol.

[41] U.S. Environemntal Protection Agency. Chemical Summary for Carbonyl Sulfide [http://www3.epa.gov/airtoxics/hlthef/carbonyl.html]. Accessed 2 Oct 2015.

[42] Interagency Testing Committee. Information Review. Carbon Oxide Sulfide; Volume Rep. no. I; 1983.

[43] National Library of Medicine. Hazardous Substances Data Bank; Bethesda, MD: HSDB; 1994. http://toxnet.nlm.nih.gov/cgi-bin/sis/htmlgen?HSDB.

[44] Szozda R (1996). Pneumoconiosis in carbon black workers. J UOEH.

[45] Occupational Safety and Health Guideline for Carbon Black (1988). National Institute for Occupational Safety and Health, Centers for Disease Control and Prevention.

[46] Grant W (1986). Toxicology of the eye.

[47] Keith S, Jones D, Rosemond Z, Ingerman L, Chappell L (2008). Toxicological profile for aluminum.

[48] Miller R, Churg A (1984). Pulmonary alveolar proteinosis and aluminum dust exposure. Am Rev Respir Dis.

[49] Goralewski G (1947). Die aluminiumlunge-eine neue gewerbeerkrankung. Z Gesamte Inn Med.

[50] McLaughlin A, Kazantzis G (1962). Pulmonary fibrosis and encephalopathy associated with the inhalation of aluminium dust. Br J Ind Med.

[51] De Vuyst P, Dumortier P (1987). Sarcoidlike lung granulomatosis induced by aluminum dusts. Am Rev Respir Dis.

[52] Tremblay C, Armstrong B (1995). Estimation of risk of developing bladder cancer among workers exposed to coal tar pitch volatiles in the primary aluminum industry. Am J Ind Med.

[53] Moffett D, Smith C, Stevens Y. Toxicological profile for barium and barium compounds. Bethesda, MD: Agency for Toxic Substances and Disease Registry 2007. http://toxnet.nlm.nih.gov/cgi-bin/sis/htmlgen?HSDB 38147518

[54] Maynard A, Kuempel E (2005). Airborne nanostructured particles and occupational health. J Nanoparticle Res.

[55] NIOSH Current Intelligence Bulletin 63: Evaluation of Health Hazard and Recommendations for Occupational Exposure to Titanium Dioxide. Bethesda, MD: National Institute for Occupational Safety and Health, Centers for Disease Control and Prevention; 2005. http://toxnet.nlm.nih.gov/cgi-bin/sis/htmlgen?HSDB.

[56] American Conference of Governmental Industrial Hygienists (2006). Documentation of the threshold limit values and biological exposure indices.

[57] NIOSH (2013). Pocket guide to chemical hazards.

[58] 91–596 PL, 1590 84 STAT., 91st Congress S 219., December 29 1970. Occupational Safety and Health Act of 1970. .

[59] World Health Organization. Ambient (Outdoor) Air Quality and Health: Fact Sheet Number 313 [http://www.who.int/mediacentre/factsheets/fs313/en/]. Accessed 2 Oct 2015.

[60] U.S Environmental Protection Agency (2012). National Ambient Air Quality Standards (NAAQS).

[61] European Commission, Directorate General for Environment. Air Quality Standards [http://ec.europa.eu/environment/air/quality/standards.htm]. Accessed 2 Oct 2015.

[62] Keith DW (2000). Geoengineering the climate: history and prospect 1. Annu Rev Energy Environ.

[63] Stenchikov G, Marquardt A, Kravitz B, Robock A. Benefits, risks, and costs of stratospheric geoengineering. Geophys Res Lett. 2009;36.

[64] Strauss J, Thomas D (1998). Health, nutrition, and economic development. J Econ Lit.

